# A Non-Inferiority Evaluation of YAHE 4.0, an Alphacypermethrin-PBO Insecticide-Treated Net Against Pyrethroid Resistant *Anopheles arabiensis* in Experimental Huts in Moshi, North-Eastern Tanzania

**DOI:** 10.3390/tropicalmed11010026

**Published:** 2026-01-18

**Authors:** Johnson Matowo, Njelembo J. Mbewe, Salum Azizi, Robert Kaaya, Oliva Moshi, Baltazari Manunda, Emmanuel Feston, Ezekia Kisengwa, Agness Msapalla, Steve Crene, Oscar Sizya, Benson Mawa, Godwin Sumari, Boniface Shirima, Silvia Mwacha, Felister Edward, Amandus Joram, Filemoni Tenu, Neema Kaaya, Naomi J. Lyimo, Franklin Mosha

**Affiliations:** 1 Pan-African Malaria Vector Research Consortium, KCMC University, Moshi P.O. Box 2240, Tanzania; salum.azizi@gmail.com (S.A.); robertkaaya@gmail.com (R.K.); olivamoshi01@gmail.com (O.M.); manundabaltazari@gmail.com (B.M.); emmafeston@gmail.com (E.F.); ezekiakisengwa2020@gmail.com (E.K.); agnessrahisa@gmail.com (A.M.); crenesteven@gmail.com (S.C.); sizya16@gmail.com (O.S.); bensonmawa3@gmail.com (B.M.); gsumari82@gmail.com (G.S.); shiribonny@gmail.com (B.S.); simonsylviamj@gmail.com (S.M.); felistadwrd@gmail.com (F.E.); aukassambili@gmail.com (A.J.); filemonitenu@gmail.com (F.T.); neemapeter11@gmail.com (N.K.); naomibongo6@gmail.com (N.J.L.); fwmosha@gmail.com (F.M.); 2 Department of Medical Parasitology and Entomology, KCMC University, Moshi P.O. Box 2240, Tanzania; 3 Department of Disease Control, London School of Hygiene and Tropical Medicine, Keppel Street, London WC1E 7HT, UK; njelembombewe@yahoo.com

**Keywords:** *Anopheles arabiensis*, blood feeding, experimental hut, insecticide-treated net, knockdown, mortality, pyrethroid resistance, susceptibility test, Tanzania

## Abstract

A new generation of insecticide-treated nets (ITNs) that incorporate the synergist piperonyl butoxide (PBO) has been shown to restore susceptibility to pyrethroids where P450 enzymes are the primary mechanism conferring the resistance. The present study evaluated the efficacy of YAHE 4.0, a PBO ITN, against wild free-flying *Anopheles arabiensis* in experimental huts in Lower Moshi, north-eastern Tanzania. It is the first evaluation of YAHE 4.0 in the country. Bio-efficacy evaluations, including susceptibility tests and cone bioassays, were conducted using the standard WHO guidelines. DuraNet Plus, a WHO-recommended PBO ITN, and Interceptor ITNs served as active and standard comparators, respectively. Unwashed and 20 times washed nets were subjected to experimental hut trials. Multiple logistic regression was employed to analyse experimental hut trial data. The results of the susceptibility testing showed that the *An. arabiensis* population of Lower Moshi was resistant to pyrethroids, but susceptible to organophosphates. Particularly, low mortality was recorded for cyhalothrin (2%) and alpha-cypermethrin (38%). Mortality rates to alpha-cypermethrin pirimiphos-methyl were 38% and 100%, respectively. The non-inferiority of YAHE 4.0 to DuraNet Plus ITN in terms of mortality and blood feeding was determined according to the WHO guidelines. The results for pooled unwashed and 20 times washed ITNs showed that YAHE 4.0 was superior to Interceptor ITN (adjusted odds ratio, AOR = 1.33; 95% CI = 1.04–1.69; non-inferiority margin, NIM = 0.68; *p*-value = 0.023) and non-inferior to DuraNet Plus (AOR = 1.02; 95% CI = 0.78–1.35; NIM = 0.72; *p*-value = 0.867) in terms of mortality. In terms of blood feeding inhibition for pooled unwashed and 20× washed ITNs, YAHE 4.0 was superior to both Interceptor ITN (AOR = 0.80; 95% CI = 0.64–1.00; NIM = 1.35; *p*-value = 0.049) and DuraNet Plus (AOR = 0.67; 95% CI = 0.52–0.86; NIM = 1.33; *p*-value = 0.002). Chemical analysis showed higher wash retention of active ingredients in YAHE 4.0 LLIN (88.9% for PBO and 94.9% for alpha-cypermethrin) compared to DuraNet Plus LLIN (89.2% for PBO and 90.5% for alphaypermethrin) before the hut trial. YAHE 4.0 LLIN demonstrated superior entomological efficacy and wash durability to DuraNet Plus and Interceptor LLINs, and fulfilled WHO non-inferiority criteria for mosquito mortality and blood-feeding inhibition. Therefore, YAHE 4.0 LLIN should be considered as an addition to the current list of pyrethroid-PBO nets used for control of pyrethroid-resistant vector populations with P450 enzymes as the main mechanism conferring resistance.

## 1. Introduction

Malaria remains an important public health problem globally [1]. However, considerable progress was made between 2000 and 2015, during which the annual deaths decreased from 841,000 to 542,000 [2]. Much of this success has been attributed to the scale-up of vector control interventions, particularly the scale-up of insecticide-treated nets (ITNs) and indoor residual spraying (IRS) [3]. Unfortunately, the success of malaria vector control in sub-Saharan Africa, which accounts for 95% of global malaria deaths, is threatened by the spread of mosquito resistance to insecticides used for insecticide-treated nets (ITNs) and indoor residual spraying (IRS) [4]. Resistance to pyrethroids and other insecticide classes has been documented in multiple regions of Tanzania [5]. Indeed, sustained malaria transmission has been recorded in areas with high resistance to multiple insecticides [6,7,8].

The next-generation ITN products (dual-active-ingredient ITNs) have been developed as an alternative tool against pyrethroid-resistant mosquitoes [9,10]. The first new class of dual-active-ingredient ITNs was treated with a mixture of a pyrethroid (PY) and a synergist, piperonyl butoxide (PBO), referred to as pyrethroid-PBO nets. PBO is a synergist that enhances insecticide toxicity by inhibiting the activity of metabolic enzymes (cytochrome P450 metabolic enzymes) that are commonly overexpressed in resistant vector populations and which often play a key role in metabolic resistance [11,12,13].

Pyrethroid-PBO nets received the WHO recommendation following cluster-RCTs in Tanzania [14] and Uganda [15], where improved efficacy of two products (Olyset^®^ Plus and PermaNet^®^ 3.0) to reduce malaria cases in areas with resistant populations of malaria vectors was documented. Moreover, pyrethroid-PBO nets are superior to standard pyrethroid-only nets in terms of mosquito mortality and blood-feeding inhibition in several experimental hut trials (EHTs) across Africa [8,16,17,18,19,20,21,22,23,24,25,26]. The WHO recommendation for pyrethroid-PBO nets was also supported by a meta-analysis of the performance of pyrethroid-PBO nets in EHTs, which was used to parameterize a malaria transmission model to predict the public health benefit of pyrethroid-PBO nets [24]. EHTs are a robust method for providing evidence of the efficacy of a new PBO product compared to a product with proven public health value [25], and the WHO requires that new candidate products be compared to the FIC product in experimental hut trials (EHTs) to assess whether the new product is not unacceptably worse (“non-inferior”) than the First in Class (FIC) based on the entomological outcome measures as a correlate of protection [26].

With metabolic resistance being a more worrying and stronger mechanism than other mechanisms such as target site mutation, there has been a drive by the industry to manufacture PBO nets. Between 2018 and 2023, seven PBO nets received full WHO recommendation, and these include Olyset^®^ Plus, PermaNet^®^3.0, Tsara Boost, Tsara Plus, Veeralin, DuraNet Plus, and Yorkool G3 [27]. During the same period, a total of 404 million PBO nets were delivered to sub-Saharan Africa, and in 2023, PBO nets accounted for 58% of the nets delivered [28]. With pyrethroid-PBO nets recently accounting for the majority of ITNs delivered to sub-Saharan Africa, an indication of high demand, potential gaps in supply could arise. Thus, there is a need to scale up manufacturing of more pyrethroid-PBO nets to meet the demand. This creates an opportunity for other pyrethroid-PBO net products to come on the market.

The WHO Global Malaria Programme has developed a procedure to decide whether a candidate ITN product should be covered by an existing policy recommendation for the vector control intervention class [29]. The procedure involves showing that the entomological efficacy of a candidate ITN demonstrates non-inferiority to the first-in-class product or another suitable active comparator [29]. The measures of entomological efficacy for an ITN are mosquito mortality and blood feeding with a set absolute difference of 7% below and above that of the active comparator, respectively [29]. The present study evaluated the efficacy of YAHE 4.0, a pyrethroid-PBO net, manufactured by the Fujian Yamei Industry & Trade Co., Ltd., against wild, free-flying, host-seeking pyrethroid-resistant *Anopheles arabiensis* in experimental huts in Lower Moshi, north-eastern Tanzania.

## 2. Materials and Methods

### 2.1. Description of the Trial Site and Hut Design

The study was carried out at Pasua field station, one of the Pan-African Malaria Vector Research Consortium (PAMVERC) field stations located in the Lower Moshi area (37°20′ E, 3°21′ S). The area is located a few kilometers from Moshi town, and 800 m above sea level, south of Mount Kilimanjaro. Most of the population in the area is engaged in agriculture with irrigated rice, and sugar cultivation is the main crop. The non-irrigated crops include maize, beans, and bananas. Two rivers, namely Njoro and Rau, provide water which is used for irrigation. Livestock in this area are mainly cattle, goats, sheep, and poultry. Irrigation activities provide an important breeding site for mosquitoes in the study area.

The type of huts where the trial was carried out is the standard traditional East African veranda trap-hut design, with brick walls plastered with mud on the inside, a wooden ceiling lined with hessian sack cloth, a corrugated iron roof, open eaves, with window traps and veranda traps on each side (Figure S1, Source: Mbewe et al. (2025), [30]. The huts are surrounded by a water-filled moat to deter the entry of scavenging ants. There are two screened veranda traps on opposite sides of the huts to capture any mosquitoes that exit via the open eaves. The eaves of the two open verandas were baffled inwardly to allow the host-seeking mosquitoes into the hut and deter exiting through the openings.

### 2.2. WHO Insecticide Susceptibility Tests

During the hut trial, the susceptibility tests were carried out using wild female *An. arabiensis* mosquitoes that emerged from the rearing of larvae which were collected from the breeding sites (rice paddies) near to experimental huts. The tests were carried out following the standard WHO protocol [31], using the test kits and insecticide-impregnated papers. Batches of 25 mosquitoes in four replicates were exposed to insecticide-impregnated papers with diagnostic concentrations of alpha-cypermethrin (0.05%), cypermethrin (0.05%), and pirimiphos-methyl (0.25%) for 1 h in WHO test kits. The knockdown effects for all tested insecticides were recorded at 10, 15, 20, 30, 40, 50, and 60 min.

A control in two replicates (50 female Anopheles mosquitoes were used for each insecticide), each with an equal number of mosquitoes, was exposed to papers impregnated with oil and run concurrently. At the end of the exposure period, mosquitoes were transferred into holding tubes (lined with untreated papers) and provided with a 10% sugar solution in cotton wool which was placed on top of the holding tube. Mortality was scored 24 h post-exposure. The susceptibility tests were carried out in a room with 25  ±  2 °C temperatures and 80  ±  10% humidity.

### 2.3. Description of the Test Product

The test item YAHE 4.0 LLIN, (Fujian Yamei Industry &Trade Co., Ltd., Fuzhou City, Fujian Province, China) is an alpha-cypermethrin pyrethroid + PBO (incorporated into polyethylene) LLIN made of monofilament yarn (120 denier), fabric weight of 37.8 g/m^2^, containing 6.6 ± 25% g/kg alpha-cypermethrin and 2.4 ± 25% g/kg PBO. The YAHE 4.0 LLIN was compared to three controls: an untreated net as a negative control, and two positive control nets, Interceptor^®^ LLINs (BASF Corporation, Florham Park, NJ, USA) and DuraNet Plus (Shobikaa Impex Private Limited, Tamil Nadu, India). Interceptor^®^ and DuraNet Plus LLINs were chosen as standard and active positive comparators, respectively. While Interceptor^®^ LLINs were impregnated with alpha-cypermethrin only, the DuraNet Plus LLINs were impregnated with both alpha-cypermethrin and PBO. Both positive control nets are recommended for use in malaria prevention and control by the WHO Prequalification Team—Vector Control (Interceptor^®^: Prequalification reference number 002-002; DuraNet Plus: Prequalification reference number 006-003). The description of the control nets is as follows: Untreated polyester nets (size: 180 cm W × 190 cm L × 150 cm H), Interceptor^®^ polyester LLIN (size: 160 cm W × 180 cm L × 180 cm H; 100D AI: 5.0 g/kg alpha-cypermethrin), fabric weight of 40 g/m^2^, and DuraNet Plus polyethylene nets (size: 160 cm W × 180 cm L × 150 cm H; 150D; AI: 6.0 g/kg alpha-cypermethrin + 2.2 g/kg PBO, fabric weight of 36 g/m^2^.

### 2.4. Preparation of Nets and Washing Procedure

Pieces of nets were cut according to the WHO Pesticide Evaluation Scheme (WHOPES) LLIN testing guideline [31]. Whole nets were washed using Savon de Marseille soap and tap water. In brief, LLINs were washed for a total of 10 min, using a wooden paddle. They were manually stirred at a rate of 20 rotations per minute for 3 min; left to soak for 4 min; then manually stirred again as before for 3 min. After washing, the LLINs were rinsed twice in 10 L of clean water following the same procedure. After rinsing, they were removed from the water and dried horizontally in the shade and stored at ambient temperature. Before the nets were sent to the Pasua experimental hut site, all nets had six holes cut in them to simulate the conditions of a damaged net following the WHO 2013 WHOPES guidelines [19]. The first series of cone bioassays was run on the unused test item and positive control net piece unwashed and washed 20 times, as well as the unwashed negative control; two replicates per net piece and five *An. gambiae s.s.* Kisumu and *An. gambiae* Muleba-Kis per replicate. Similarly, two replicates per net piece and five *An. arabiensis* KGB and wild F1 *An. arabiensis* per replicate. In total, 50 mosquitoes of each strain were used to test each unwashed and 20 times washed piece of the untreated net, Interceptor^®^ LLIN, DuraNet Plus LLIN, and YAHE 4.0 LLIN.

The second series of cone bioassays was run on net pieces acquired from hut-used unwashed and 20 times washed test items and positive control nets as well as an unwashed negative control. Similar replicates per net piece, as well as the number and strain of mosquitoes, to the first series of cone bioassays were used. In total, 50 mosquitoes of each of the four strains were used to test each hut-used unwashed and washed Interceptor^®^ LLIN, DuraNet Plus LLIN, and YAHE 4.0 LLIN. Cone bioassays followed the standard WHO procedures [32]. Five 3-day-old female mosquitoes (sugar-fed) were introduced into each cone and exposed for 3 min. Mean knockdown at 60 min post-exposure and mortality at 24 h post-exposure was calculated for each net. A mosquito was classified as knocked down or dead if it was immobile or unable to stand or take off, or flew in an uncoordinated manner at 60 min or 24 h, respectively.

### 2.5. Experimental Hut Trial

Washed and unwashed candidate LLINs were evaluated using seven East African experimental huts for their effects on the wild pyrethroid-resistant *An. arabiensis* mosquitoes.

The following seven treatment arms were compared:

1. Unwashed YAHE 4.0 LLIN
2. Unwashed Interceptor^®^ LLIN
3. Unwashed DuraNet Plus
4. YAHE 4.0 LLIN washed 20 times
5. Interceptor^®^ LLIN washed 20 times
6. DuraNet Plus LLIN washed 20 times
7. Untreated polyester net

Treatment arms were listed from 1 to 7, then, using https://hamsterandwheel.com/grids/index2d.php, the treatments were randomly put in a Latin square. Seven nets were used per treatment arm, and each of the seven nets was tested for one night during the seven consecutive nights. At the end of the seven nights, the huts were carefully cleaned and aired to remove potential contamination. The treatment arm was then rotated to a different hut to account for possible location bias, i.e., differences between huts.

In this study, cows were used as bait instead of humans because the predominant malaria vector at the study site is *An. arabiensis*, which has shown zoophilic feeding behaviour as reported in a previous study by Mahande et al. (2007) [33]. To account for individual cow attractiveness to mosquitoes, seven cows were also rotated daily between the seven experimental huts using a Latin square design. The study was performed for seven rounds over seven weeks to ensure complete rotation through the huts. In the present experimental hut trial study, we used the JD Challenger method, found at https://github.com/JDChallenger/WHO_NI_Tutorial, to assess whether the study had sufficient power. Proportional outcomes, including mortality and blood-feeding, were compared using mixed effects logistic regression where huts, cows, net replicates, and nightly observational error were included as random or fixed effects.

The primary outcomes measured in experimental huts were:

1. Deterrence (reduction in hut entry relative to the control huts fitted with untreated nets)
2. Induced exiting (the proportion of mosquitoes that are found in exit traps and veranda relative to control)
3. Blood-feeding inhibition (the reduction in blood-feeding relative to the control)
4. Mortality (the proportion of mosquitoes killed)
5. Personal protection, which can be estimated as follows:
  Personal protection (%) = 100 (Bu − Bt)/Bu, where Bu is the total number of blood-fed in the huts with untreated nets, and Bt is the total number of blood-fed in the huts with LLIN-treated nets
6. The overall killing effect of the treatment was estimated as follows:
  Insecticidal effect (%) = 100 (Kt − Ku)/Tu, where Kt is the number killed in the huts with LLIN-treated nets, Ku is the number dying in the huts with untreated nets, and Tu is the total collected from the huts with untreated nets.

### 2.6. Chemical Analysis

At the conclusion of the hut study, a total of 60 pieces of netting, 30 × 30 cm in size, used in cone bioassays, individually wrapped in aluminium foil, labelled with the test or reference item code on the outside were shipped to Walloon Agricultural Research Centre (CRA-W), Carson Building, RueduBordia, 11, B-5030–Gembloux, Belgium, for chemical analysis. These net pieces include 20 pieces of each the Interceptor^®^ LLINs, DuraNet Plus LLINs, and YAHE 4.0 LLINs as summarized in Table S1.

The alpha-cypermethrin and piperonyl butoxide content in net samples of YAHE^®^ 4.0 and DuraNet^®^ Plus was determined using the combined CRA-W internal methods PA-U10-RESSM016 and PA-U10-RESSM026 with extraction with *n*-heptane using dicyclohexyl phthalate as internal standard and determination by Gas Chromatography with Flame Ionisation Detection (GC-FID). The alpha-cypermethrin content in net samples of Interceptor^®^ was determined using the adapted CIPAC 454/LLIN/M2/3 with extraction with *n*-heptane using dicyclohexyl phthalate as internal standard and determination by Gas Chromatography with Flame Ionisation Detection (GC-FID). The fabric weight (mass of net per m^2^) was determined on net samples using the CRA-W method PA-U10-NET001 based on the ISO 3801 method to convert the active substance content from g/kg into mg/m^2^. The performance of the analytical methods was controlled during the analysis of samples to validate the analytical results.

### 2.7. Data Analysis

Statistical analysis was done using STATA version 16.1. Proportions of mosquitoes knocked down and dead were calculated for all experiments. Logistic regression was used for proportional data (adjusting for the effect of hut and cow) and Poisson regression for numerical data. The odds ratios obtained from a logistic regression were used to determine the non-inferiority of the test item to the positive control (DuraNet Plus LLIN/Interceptor^®^ LLIN). Variance estimates were adjusted for clustering by each hut on the night of collection. The primary criteria in the evaluation were blood-feeding inhibition and mortality. Non-inferiority analysis was conducted following WHO guidelines, taking an odds ratio corresponding to 7% non-inferiority margin [29]. The results of the susceptibility tests were evaluated as recommended by WHO criteria [31], as follows: 98–100% mortality indicates susceptibility, 90–97% mortality indicates a possible resistance candidate, and less than 90% mortality suggests resistance.

## 3. Results

### 3.1. WHO Susceptibility Tests

**Figure 1:**
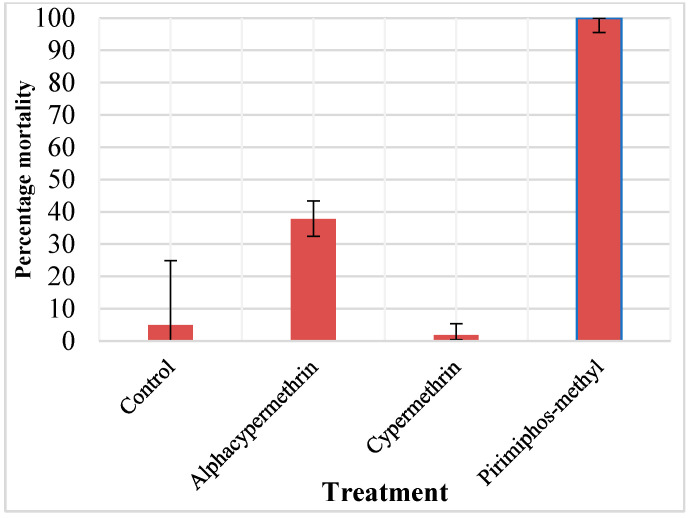
Mortality rates of wild *An. arabiensis*, reared as larvae from experimental hut station during the trial, to alpha-cypermethrin, cypermethrin and pirimiphos-methyl.

Mortality of the wild pyrethroid-resistant *An. arabiensis* from Lower Moshi exposed to 0.05% alpha-cypermethrin, and 0.05% cypermethrin in WHO susceptibility tests was 38% and 2%, respectively, indicating frequency of resistance to alpha-cypermethrin, and cypermethrin of 62% and 98%, respectively, at the study site. Mortality of the wild pyrethroid-resistant *An. arabiensis* from Lower Moshi exposed to 0.25% primiphos-methyl was 100%. Control mortality of the *An. arabiensis* exposed to untreated paper was 5%. (Figure 1).

### 3.2. Experimental Hut Trial Results

#### 3.2.1. Number of Mosquitoes Collected from Huts and Exiting Rates

A total of 2901 female *An. arabiensis* were collected over a period of 112 nights. The average (geometric mean) number of *An. arabiensis* mosquitoes per treatment arm per night ranged from 2.6 to 6.3, and all trial arms recorded high *An. arabiensis* exiting rates of >82% (Table 1).

#### 3.2.2. Blood-Feeding

The blood-feeding rates for YAHE 4.0 nets (32.1% and 32.8%) before and after being washed 20 times, respectively, were lower than other treatments except for the Interceptor LLIN washed 20 times which induced a blood-feeding of 30.4% (Table 1). Blood-feeding rates recorded in all treatment arms were significantly lower than the untreated control arm, except for washed DuraNet Plus (AOR = 1.0; 95% CI = 0.7–1.3; *p* = 0.765; Z = −0.30). Blood feeding inhibition was highest in the washed Interceptor arm followed by the unwashed and washed YAHE 4.0 arms (Table 1). The non-inferiority analysis showed that the unwashed YAHE 4.0 was non-inferior and superior to the unwashed Interceptor, while the washed YAHE 4.0 was non-inferior to the washed Interceptor in terms of blood feeding. Furthermore, the unwashed YAHE 4.0 was non-inferior to the unwashed DuraNet Plus, while the washed YAHE 4.0 was superior to the washed DuraNet Plus (Table 2). Pooled unwashed and washed non-inferiority analysis showed that YAHE 4.0 was non-inferior and superior to both Interceptor (AOR = 0.80; 95% CI = 0.64–1.00; non-inferiority margin, NIM = 1.35; *p*-value = 0.049) and DuraNet Plus (OR = 0.67; 95% CI = 0.52–0.86; NIM = 1.33; *p*-value = 0.002) in terms of blood feeding.

#### 3.2.3. Mortality

The likelihood of *An. arabiensis* mortality in all treated arms was significant (AOR ≥ 17.7; *p* < 0.001), ranging from 44.1% to 55.7% compared to the mortality of the control arm (4.5%) (Table 1). The percentage of mortality recorded for unwashed YAHE 4.0 LLIN was 46.4% (41.1–51.6%) while that of 20 times washed YAHE 4.0 LLIN was higher, 49.7% (44.3–55.1). However, the percentage of mortality recorded for DuraNet Plus unwashed and 20 times washed was slightly higher than that of the YAHE 4.0 LLIN 54.0% (48.2–59.8%) and 55.7% (50.6–60.7%), respectively, while that of the Interceptor LLIN washed 20 times was higher than that of YAHE 4.0 and DuraNet Plus LLLINs washed 20 times (Table 1). In terms of mortality, the non-inferiority analysis showed that the unwashed and washed YAHE 4.0 were non-inferior to the unwashed and washed Interceptor, respectively (Table 3). Unwashed YAHE 4.0 did not show non-inferiority to unwashed DuraNet Plus, while washed YAHE 4.0 was non-inferior and superior to washed DuraNet Plus (Table 3). Pooled unwashed and washed non-inferiority analysis showed that YAHE 4.0 was non-inferior and superior to Interceptor (AOR = 1.33; 95% CI = 1.04–1.69; NIM = 0.68; *p*-value = 0.023) and non-inferior to DuraNet Plus (AOR = 1.02; 95% CI = 0.78–1.35; NIM = 0.72; *p*-value = 0.867) in terms of mortality.

### 3.3. Supplementary Laboratory Bioassays

#### 3.3.1. WHO Cone Bioassay Tests Before the Hut Trial

**Figure 2:**
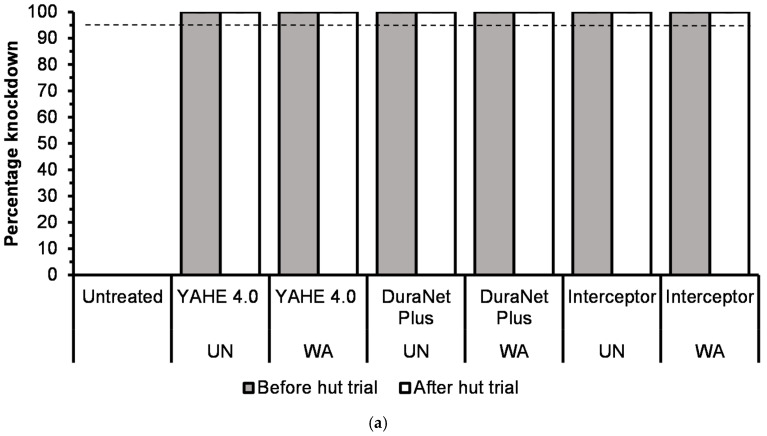

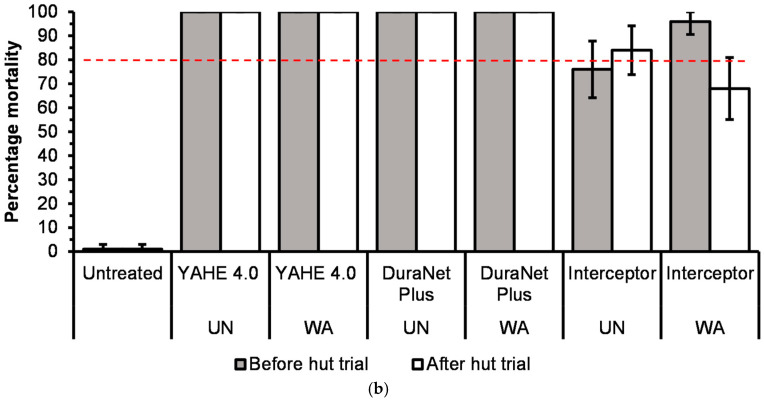
(**a**) Knockdown rates during cone bioassays before hut trial and after experimental hut trials using laboratory-susceptible *An. gambiae s.s* Kisumu strain (UTN = Untreated net; UN = Unwashed; WA = Washed). (**b**) Mortality rates during cone bioassays before the hut trial and after the hut trial using Kisumu strain UTN = Untreated net; UN = Unwashed; WA = Washed). Red dash line represents the WHO bi-efficacy cut-off of mortality while the black dash line represents the WHO bio-efficacy cut-off of knockdown.

**Figure 3:**
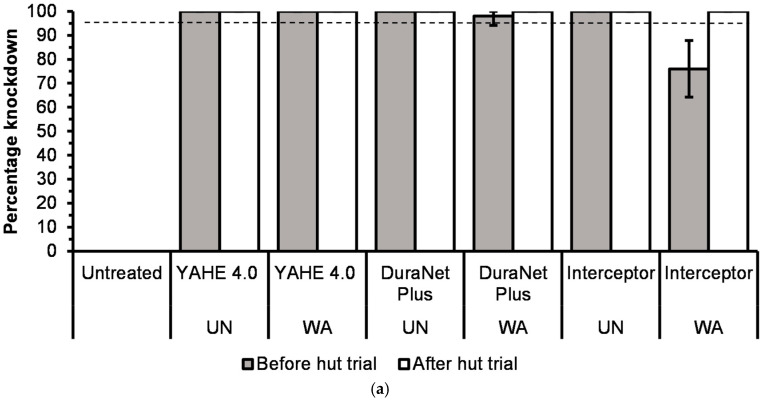

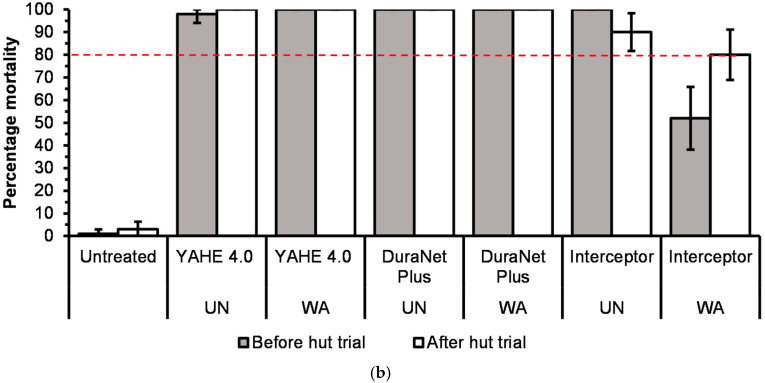
(**a**) Knockdown rates during cone bioassays before hut trial and after experimental hut trials using laboratory-susceptible *An. arabiensis* KGB strain (UTN = Untreated net; UN = Unwashed; WA = Washed). (**b**) Mortality rates during cone bioassays before the hut trial and after the hut trial using susceptible *An. arabiensis* KGB strain (UTN = Untreated net; UN = Unwashed; WA = Washed). Red dash line represents the WHO bi-efficacy cut-off of mortality while the black dash line represents the WHO bio-efficacy cut-off of knockdown.

**Figure 4:**
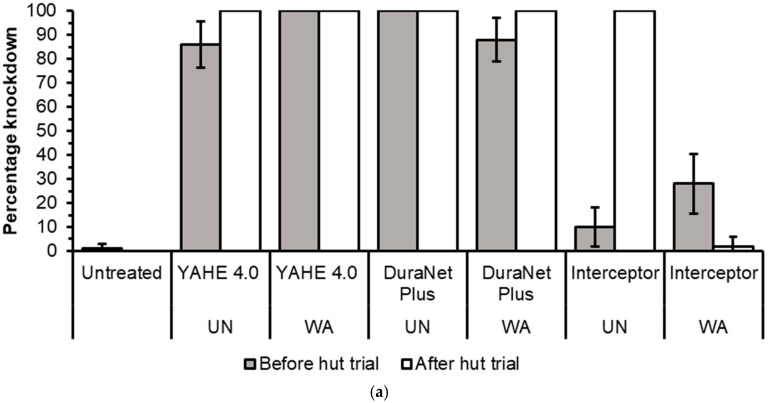

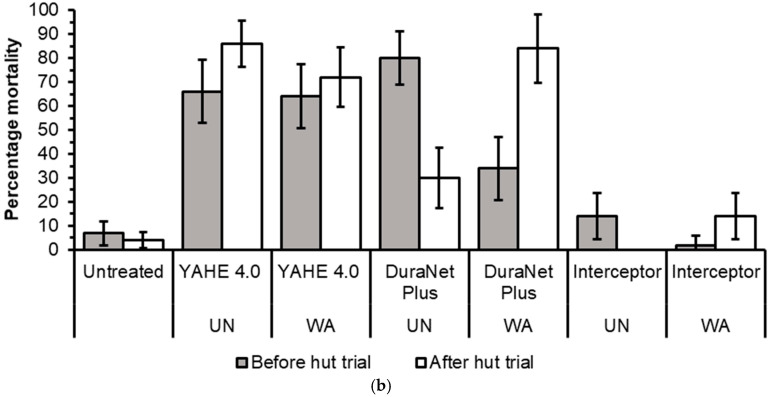
(**a**) Knockdown rates during cone bioassays before hut trial and after experimental hut trials using laboratory-reared pyrethroid-resistant *An. gambiae s.s* Muleba-Kis strain (UTN = Untreated net; UN = Unwashed; WA = Washed). (**b**) Mortality rates during cone bioassays before the hut trial and after the hut trial using laboratory-reared pyrethroid-resistant Muleba-Kis strain UTN = Untreated net; UN = Unwashed; WA = Washed).

**Figure 5:**
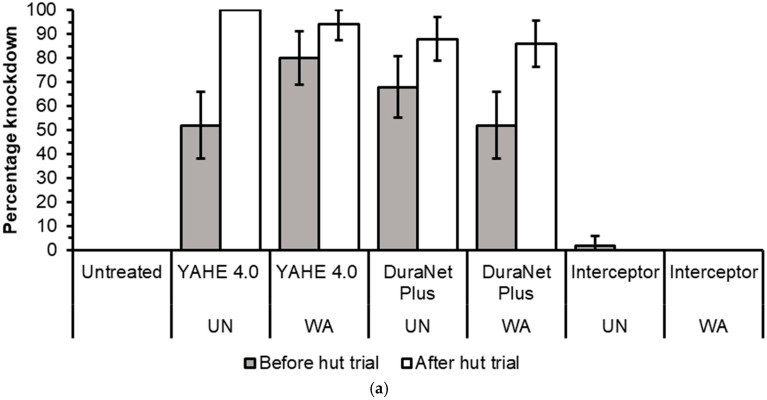

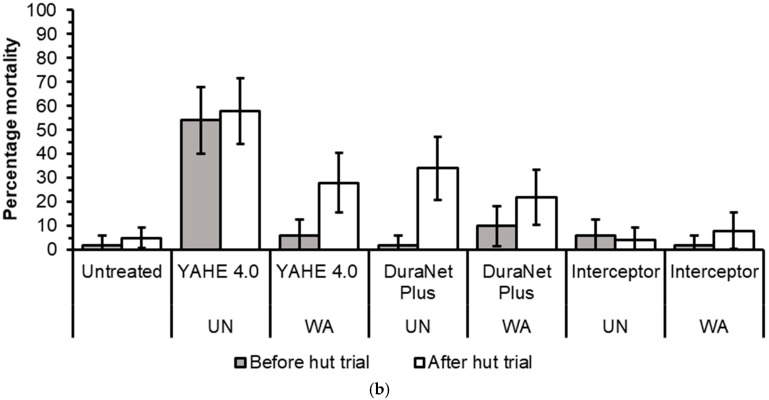
(**a**) Knockdown rates during cone bioassays before hut trial and after experimental hut trials using wild pyrethroid-resistant *An. arabiensis* from the study area (UTN = Untreated net; UN = Unwashed; WA = Washed). (**b**) Mortality rates during cone bioassays before the hut trial and after the hut trial using wild pyrethroid-resistant *An. arabiensis* from the study area (UTN = Untreated net; UN = Unwashed; WA = Washed).

Before the hut trial, the 1 h knockdown was 100% for all treatment-susceptible strains except for DuraNet Plus washed 20 times (98%), for both Kisumu and KGB strains, and Interceptor washed 20 times (76%) on the KGB strain (Figure 2a and Figure 3a).Low to high knockdown was recorded for pyrethroid-resistant strains of Muleba-Kis and wild *Anopheles arabiensis* across all LLIN types, ranging from 0% for washed Interceptor LLIN on Muleba-Kis to 100% for both unwashed DuraNet Plus on wild *An. arabiensis* and 20 times washed YAHE 4.0 LLIN on Muleba-Kis (Figure 4a and Figure 5a). For susceptible strains, the mortality rates were 100%, except for Interceptor LLINs, unwashed and washed, which induced mortality rates of less than 100%. (Figure 2b and Figure 3b). For Muleba-Kis, low to moderate mortality rates were recorded for resistant strains, ranging from 2% (−0.3–6.5%) for washed interceptor LLIN to 80% (64.4–96.5%) for unwashed DuraNet Plus LLIN. However, the mortality rate for the unwashed DuraNet Plus LLIN was not significantly different from that of unwashed YAHE 4.0 LLIN, 66% (50.8–81.1%), and that of washed YAHE 4.0 LLIN, 64% (46.4–81.5%) (Figure 4b). For the wild *An. arabiensis*, very low mortality rates were recorded except for unwashed YAHE 4.0 LLIN, which induced a mortality rate of 54% (34.9–73.1%). (Figure 5b).

#### 3.3.2. WHO Cone Bioassays Tests After the Hut Trial

After the hut trial, the 1 h knockdown was 100% for all treatments with both susceptible strains (Kisumu and KGB) (Figure 2a and Figure 3a). This was the same for the resistant Muleba-Kis strain that induced 100% knockdown, except for the washed Interceptor LLIN. For resistant wild *An. arabiensis*, there was no significant difference between the knockdown rates of washed and unwashed LLINs. However, the highest knockdown rates were recorded for YAHE LLINs, while the moderate knockdown rates were recorded for the DuraNet Plus LLINs. The knockdown rates for Interceptor LLINs were 0% with wild *An. arabiensis* (Figure 4a and Figure 5a).

The mortality rate was 100% for susceptible strains, except Interceptor LLINs, which induced mortality rates of 84% and 68% with the Kisumu strain for unwashed and washed nets, respectively. For the susceptible KGB strain, Interceptor LLIN induced higher mortality rates of 90% for unwashed net and 80% for washed net. (Figure 2b and Figure 3b). For resistant strains, the mortality rates were generally less than 90%. For Muleba-Kis, the highest mortality rate was recorded for the YAHE 4.0 unwashed LLIN, while the lowest was recorded for the Interceptor unwashed LLIN. There was no significant difference between the mortality rates of Muleba-Kis exposed to washed YAHE 4.0 LLIN 72% (44.8–99.1%) and washed DuraNet Plus 84% (39.6–128.4%). However, there was a significant difference between the mortality rates of Muleba-Kis exposed to unwashed YAHE 4.0 LLIN 86% (74.2–97.8%) and unwashed DuraNet Plus 30% (19.9–40.1%). For the pyrethroid-resistant wild *An. arabiensis*, the mortality rates remained consistently low after the trial, ranging from 4% to 8% for interceptor LLINs to 58% for unwashed YAHE 4.0 LLIN (Figure 4b and Figure 5b).

### 3.4. Chemical Analysis

The analytical results regarding the determination of the active substance/synergist content on incorporated net samples of YAHE^®^4.0 LLIN and DuraNet^®^Plus LLIN and on coated net sample Interceptor^®^LLIN are summarized in Supplementary Material Table S2a,b, Table S3a,b and Table S4, respectively. The tables summarize the amounts of the active substance (alpha-cypermethrin) and synergist (PBO) before washing, after 20 washes, before and after the hut trial.

The initial amount of alpha-cypermethrin in the YAHE^®^ 4.0 LLIN before washing and before the trial was 8.70 g/kg. Following 20 times washing, the amount dropped to 8.25 g/kg. After the trial, the amount of alpha-cypermethrin in unwashed YAHE^®^ 4.0 LLIN was 8.33 g/kg, slightly lower than the amount that was observed before the trial, while the amount of alpha-cypermethrin after 20 washes was 7.75 g/kg (Table S2a). Likewise, the amount of PBO in the YAHE^®^ 4.0 LLIN before washing and before the trial was found to be 3.46 g/kg. Once the net was washed 20 times, the amount dropped to 3.08 g/kg. After the trial, the amount of PBO in unwashed YAHE^®^ 4.0 LLIN was 3.11 g/kg, slightly lower than the amount that was observed before the trial, while the amount of PBO after 20 washes was 2.73g/kg (Table S2b). Also, the YAHE^®^ 4.0 LLIN had a very high wash resistance index to alpha-cypermethrin (~100%) with very high retention of 94.9% and 93% before the hut trial and after the hut trial, respectively (Table S2a). The wash resistance index for the PBO was also very high, i.e., 99.4% before the hut trial and 99.3% after the hut trial. However, the retention of PBO was slightly lower than that of alpha-cypermethrin, 88.9% before the hut trial and 87.7% after the hut trial (Table S2b).

For the active positive comparator, the DuraNet^®^ Plus LLIN, the initial amount of alpha-cypermethrin before washing and before the trial was 6.11 g/kg. After 20 washes, the amount dropped to 5.53 g/kg. After the trial, the amount of alpha-cypermethrin in unwashed DuraNet^®^ Plus LLIN was 5.91 g/kg, slightly lower than the amount that was observed before the trial, while the amount of alpha-cypermethrin after 20 washes was 5.55 g/kg (Table S3a). Likewise, the amount of PBO in the DuraNet^®^ Plus LLIN before washing and before the trial was 1.86 g/kg, dropping to, 1.66 g/kg after 20 washes. After the trial, the amount of PBO in unwashed DuraNet^®^ Plus LLIN was 1.71 g/kg, slightly lower than the amount that was observed before the trial, while the amount of PBO after 20 washes was 1.61 g/kg (Table S3b). For the active positive comparator, the DuraNet^®^ Plus LLIN, the wash resistance index for alpha-cypermethrin was also very high (~100%), with its retention being slightly lower than that of alpha-cypermethrin in the YAHE^®^ 4.0 LLIN (90.5% and 93.9% before the hut trial and after the hut trial, respectively). Likewise, the wash resistance index to PBO in the DuraNet^®^ Plus LLIN was approximately 100% as for the YAHE^®^ 4.0 LLIN. However, retention of PBO in the DuraNet^®^ Plus LLIN was 89.2% and 93.9% before the hut trial and after the hut trial, respectively (Table S3b).

For the standard positive comparator, the Interceptor^®^ LLIN, the initial amount of apha-cypermethrin before washing and before the trial was 5.25 g/kg. After 20 washes, the amount dropped significantly to 2.09 g/kg. After the trial, the amount of alpha-cypermethrin in the unwashed nets had already dropped significantly to 3.23 g/kg, compared to the amount that was observed before the trial, while the amount of alpha-cypermethrin after 20 washes was 1.03 g/kg only (Table S4). For the standard positive comparator, the Interceptor^®^ LLIN, the wash resistance index for alpha-cypermethrin was 95.5% and 94.4% before the hut trial and after the hut trial, respectively, while its retention was significantly lower, 39.9% and 31.8% (Table S4), than that of YAHE^®^ 4.0 LLIN.

## 4. Discussion

Pyrethroid–PBO LLINs are recommended for the control of pyrethroid-resistant mosquitoes because the addition of Piperonyl Butoxide (PBO) to pyrethroid nets imposes a blockade on metabolic resistance mechanisms in these mosquitoes, thereby partially restoring susceptibility to pyrethroid [14,17,34,35]. Several experimental hut trials across Africa PBO LLINs have demonstrated PBO LLINs to be a strategy to overcome pyrethroid resistance, especially in areas where resistance is driven by overexpression of P450 enzymes known to metabolize pyrethroids [17,19,27,35,36]. The Olyset^®^ Plus LLINs are the First in Class (FIC) LLINs that received a World WHO policy recommendation [32], following their demonstrated impact on malaria reduction in cluster randomized trials [14,15]. However, there is a need for more PBO nets on the market [14], and other manufacturers of LLINs have produced pyrethroid-PBO nets that differ from Olyset^®^ Plus in terms of design and specifications. With a gradual increase in demand in sub-Saharan Africa, having more PBO-ITN products will not only contribute to the sustainability of supply but also provide options of PBO-ITN sources for National Malaria Control Programs. Timely supply of PBO-ITNs is crucial for National Malaria Control Programs to sustain universal access and coverage.

The present study was designed to investigate the efficacy of the YAHE 4.0 LLIN (alpha-cypermethrin & PBO treated LLIN), after twenty washes according to the WHO standardized washing procedure [36,37], in an area dominated by *An. arabiensis* [38,39,40,41,42,43,44]. Results of the susceptibility tests have confirmed the high-level resistance to alpha-cypermethrin and cypermethrin insecticides in *An. arabiensis* population in the study area. Previous studies in the same study area by Matowo et al. (2010, 2014) [38,39] reported resistance of the *An. arabiensis* to other pyrethroids, including permethrin, deltamethrin and lambda cyhalothrin, but susceptible to pirimiphos-methyl (organophosphate). Therefore, similar to the previous studies, the *An. arabiensis* in the study area remains susceptible to pirimiphos-methyl.

According to the WHO cone bioassays that were conducted against susceptible *An. gambiae s.s.* Kisumu and *An. arabiensis*-KBG strains before washing, and after 20 washes; before and after hut trials, all LLINs met WHO cut-offs of >80% mortality or >95% knockdown before and after the hut trial, except the knockdown for Interceptor LLIN washed 20 times on the KGB strain before the trial. However, after the trial, the 20 times washed knocked down 100% of the KGB strain.

For resistant strains, before the trial, the WHO cut-off of >95% knockdown was met by the unwashed YAHE 4.0 LLIN and unwashed DuraNet Plus for Muleba-Kis. After the trial, the WHO cut-off of >95% knockdown was met by unwashed and 20 times washed YAHE 4.0 LLIN, unwashed and 20 times washed DuraNet Plus LLIN, unwashed Interceptor LLIN on Muleba-Kis, and unwashed YAHE LLIN on the wild *An. arabiensis* from the study area. The WHO cut-off of >80% mortality was met by unwashed DuraNet Plus before the hut trial; unwashed YAHE 4.0 LLIN, unwashed DuraNet Plus, and 20 times washed DuraNet Plus after the trial for the Muleba-Kis. None of the LLINs met the WHO cut-off of >80% mortality on the resistant wild *An. arabiensis*. However, unwashed YAHE 4.0 LLIN induced the highest mortality in the wild *An. arabiensis* before and after the trial.

All LLINs (unwashed and washed 20 times) had similar exophily rates. However, the pyrethroid nets with PBO (YAHE 4.0 and DuraNet Plus) and pyrethroid-only LLIN (Interceptor) induced a higher exophily than that of Veeralin LLIN, which was tested elsewhere in Tanzania against PermaNet 3.0 and DuraNet LLINs [45]. Veeralin is another alpha-cypermethrin and PBO LLIN that was recently evaluated in Tanzania [45], while PermaNet 3.0 is another type of pyrethroid-PBO net that contains deltamethrin instead of alpha-cypermethrin. In another study by Kweka et al. (2019) [46], the exophily for DuraNet was 78.4%, similar to the exophily induced by the Interceptor LLIN in this study. However, for DuraNet, the alphacypermethrin is incorporated into the fibers, while for Interceptor LLIN, the alpha-cypermethrin is surface-coated.

In this study, YAHE 4.0 LLIN induced significantly high blood-feeding inhibition compared to other types of LLINs. However, this was lower than that of Veeralin LLIN [45]. Such a difference could be because the present study was conducted in an area where *An. arabiensis* is predominant, while Veeralin was tested against *An. funestus*. The two species differ in terms of behaviour.

In terms of mortality, YAHE 4.0 LLIN washed 20 times was superior to DuraNet Plus, inducing higher mortality against the wild free-flying pyrethroid-resistant *An. arabiensis* mosquitoes. This finding is consistent with the findings of the recent studies conducted elsewhere in Tanzania, and Benin where Vector Guard^®^ (a new alpha-cypermethrin-PBO LLN) was superior to Olyset^®^ Plus nets in terms of mortality [23,47]. Elsewhere in Tanzania, significantly higher mortalities of pyrethroid-resistant *An. funestus* (s.l.) were recorded by the unwashed and 20 times washed Veeralin compared to DuraNet LLIN in the experimental hut, where the incorporation of PBO in Veeralin showed increased mortality impact than in non-PBO DuraNet LLIN [21]. Similar killing effect of Veeralin against pyrethroid-resistant mosquitoes has been recorded elsewhere in Côte d’Ivoire [20]. The findings of the present study support the findings of the previous few studies that an alpha-cypermethrin–PBO net could also be effective in controlling pyrethroid-resistant malaria vectors. It also adds to the large body of evidence that pyrethroid-PBO–PBO nets are more efficacious than pyrethroid-only ITNs against mosquitoes with resistance mediated by mixed-function oxidases. A candidate LLIN meets the WHO PQT phase II efficacy criteria if it performs as well as or better than the reference LLIN when washed 20 times in terms of blood-feeding inhibition and mortality. In the present study, the maximum mortality that was induced by YAHE 4.0 LLIN was 74.9%, implying that other metabolic enzymes may be responsible for the observed pyrethroid resistance, as previously reported by Matowo et al. (2010) [37]. Indeed, pre-exposure to *An. arabiensis* to PBO followed by exposure to a diagnostic dose of permethrin in CDC bottle bioassays resulted in partial restoration of the susceptibility, implying involvement of other resistance mechanisms, especially the β-esterases [38].

Based on the parameters measured, Interceptor net performed worse than the other LLINs tested. In an area with pyrethroid-resistant malaria vectors, YAHE 4.0 LLIN with PBO can provide additional protection in terms of reducing blood feeding and increasing mosquito mortality, compared to a pyrethroid-only net.

For the chemical analysis, the amounts of alpha-cypermethrin and PBO in the YAHE^®^ 4.0 LLIN seem to be well above the declared amounts, and equivalent to the amounts that were claimed by the manufacturer. The declared (nominal) active ingredient and synergist content were 6.25 g/kg ± 25% alpha-cypermethrin and 2.2 g/kg ± 25% Piperonyl butoxide with 38 g/m^2^ for the density. The results also show that amounts of alpha-cypermethrin and PBO in the YAHE^®^ 4.0 LLIN are much higher compared to the amounts of alpha-cypermethrin and PBO in the active positive comparator, the DuraNet^®^ Plus LLIN. However, the amount of alpha-cypermethrin in the standard net, the Interceptor^®^ LLIN, was even lower than that of DuraNet^®^ Plus LLIN, and it decreased significantly with time and washing compared to the YAHE^®^ 4.0 and DuraNet^®^ Plus LLINs. In general, the active ingredient (AI) content from 0 to 20 washes met the WHO criteria of the AI content ±25% of the claim for the YAHE^®^ 4.0 and DuraNet^®^ Plus LLINs. The standard Interceptor^®^ LLIN did not meet the WHO criteria of the AI content ±25%, probably because, unlike the two LLINs with the AIs incorporated into polyethylene, the alpha-cypermethrin is coated onto the polyester of the Interceptor^®^ LLIN. The amounts of active ingredients noted for the YAHE 4.0 LLIN after analysis are different from those of previous studies [48,49] and as reported in WHOPES reports [50,51,52,53,54].

## 5. Study Limitations

The present study was based on a semi-field phase II experimental hut trial; hence, no parasitological or clinical impact assessment was carried out. Nevertheless, in the experimental hut trials, the entomological outcome measures are a correlate of human protection from malaria transmission. For the Standard Comparator, we used Interceptor LLIN with a surface coating of alphacypermethrin because the standard LLINs with alphacypermethrin incorporated into the fibers, such as DuraNet, MAGNet LLIN, and MiraNet LLIN (which would match the test net and the active comparator), were not available.

## 6. Conclusions

The findings of this study have shown that blood-feeding rates recorded for 20 times washed YAHE 4.0 LLIN were statistically similar to those of the 20 times washed PQT-approved PBO/pyrethroid (DuraNet Plus) and a positive control in this trial. Also, the mortality induced by 20 times washed YAHE 4.0 LLIN was statistically similar to that recorded for the 20 times washed DuraNet Plus LLIN. In addition, the mortality and knockdown rates for the YAHE 4.0 LLIN against susceptible strains exceeded the WHO bio-efficacy cut-offs of ≥80% mortality or ≥95% knockdown. This provides evidence that YAHE 4.0 LLIN has met WHO PQT efficacy criteria for long-lasting insecticidal nets (LLINs). Higher wash retention of active ingredients (PBO and alpha-cypermethrin) were recorded in YAHE 4.0 compared to DuraNet Plus before and after the hut trial. Based on the bio-efficacy and wash resistance findings of the YAHE LLIN, we recommend YAHE 4.0 LLIN to be considered a suitable tool for protection against pyrethroid-resistant malaria vectors and should be added to the pool of PBO LLLINs. The addition of YAHE 4.0 LLIN to the WHO-recommended pyrethroid-PBO nets will enable malaria control programs to select cost-effective ITNs, improving access to effective protection from malaria in areas where malaria vectors are resistant to pyrethroids. However, large-scale trials, especially the cluster randomized controlled trials in the community are needed before full WHO recommendations can be considered.

## Figures and Tables

**Table 1:** Exiting, blood feeding, and mortality of wild free-flying *Anopheles arabiensis* in experimental huts.

Variables	Type of LLIN						
	Untreated	YAHE 4.0	YAHE 4.0	Interceptor	Interceptor	DuraNet Plus	DuraNet Plus
No of washes	0	0	20	0	20	0	20
Total females caught	374	343	326	492	709	287	370
Mean females caught/night	3.3	3.1	2.9	4.4	6.3	2.6	3.3
Total females exited	312	284	268	424	594	248	304
% Exophily (95% CI)	83.4 (79.6–87.1)	82.8 (78.8–86.8)	82.2 (78.1–86.4)	86.2 (83.1–89.2)	83.8 (81.1–86.5)	86.4 (82.4–90.4)	82.2 (78.3–86.1)
Total females blood fed	171	110	107	188	216	101	167
% Blood fed (95% CI)	45.7 (40.6–50.8)	32.1 (27.1–37.0)	32.8 (27.7–37.9)	38.2 (33.9–42.5)	30.4 (27.1–33.8)	35.2 (29.7–40.7)	45.1 (40.1–50.2)
Blood feeding AOR (95% CI) *p*-value; Z-value	1.0	0.5 (0.3–0.7) **<0.001**; −4.19	0.6 (0.4–0.8) **0.001**; −3.22	0.7 (0.5–1.0) **0.044**; −2.02	0.6 (0.5–0.8) **0.001**; −3.28	0.6 (0.4–0.9) **0.009**; −2.62	1.0 (0.7–1.3) **0.765**; −0.30
% Blood feeding inhibition	0	29.9	28.2	16.4	33.4	23.0	1.3
Total females dead	17	159	162	238	313	155	206
% Mortality (95% CI)	4.5 (2.4–6.6)	46.4 (41.1–51.6)	49.7 (44.3–55.1)	48.4 (44.0–52.8)	44.1 (40.5–47.8)	54.0 (48.2–59.8)	55.7 (50.6–60.7)
Mortality AOR (95% CI) *p*-value; Z-value	1	66.9 (22.7–197.4) **<0.001**; 7.62	74.9 (25.4–220.9) **<0.001**; 7.82	51.9 (17.7–151.8) **<0.001**; 7.21	54.6 (18.7–159.1) **<0.001**; 7.32	83.1 (27.9–247.1) **<0.001**; 7.95	60.6 (20.6–178.3) **<0.001**; 7.45
% Overall killing effect	0	38.0	38.8	59.1	79.1	36.9	50.5

CI: Confidence interval; AOR: Adjusted odds ratio; *p*-values in bold are significant.

**Table 2:** Non-inferiority analysis of unwashed and washed Interceptor, DuraNet Plus, and YAHE 4.0 LLINs in terms of blood feeding.

Outcome	Reference Net	Candidate Net	AOR	95% CI (*p*-Value, Z-Value)	Non Inferiority Margin	Test Outcome
Primary: Mortality (72 h)	Unwashed Interceptor ™	Unwashed YAHE 4.0	0.66	0.48–0.92 (0.014, −2.47)	1.33	Non-inferior and superior
	Washed Interceptor ™	Unwashed YAHE 4.0	0.93	0.68–1.27 (0.665, −0.43)	1.37	Non-inferior and not superior
	Unwashed DuraNet Plus	Unwashed YAHE 4.0	0.78	0.54–1.13 (0.194, −1.30)	1.34	Non-inferior and not superior
	Washed DuraNet Plus	Washed YAHE 4.0	0.64	0.47–0.85 (0.004, −2.91)	1.32	Non-inferior and superior

**Table 3:** Non-inferiority analysis of unwashed and washed Interceptor, DuraNet Plus, and YAHE 4.0 LLINs in terms of mortality.

Outcome	Reference Net	Candidate Net	OR	95% CI (*p*-Value, Z-Value)	Non Inferiority Margin	Target Outcome	Test Outcome
Primary: Mortality (72 h)	Unwashed Interceptor ™	Unwashed YAHE 4.0	1.29	0.90–1.84 (0.161, 1.40)	0.75	Superiority	Non-inferior and not superior
	Washed Interceptor ™	Unwashed YAHE 4.0	1.37	0.98–1.91 (0.061, 1.87)	0.75	Superiority	Non-inferior and not superior
	Unwashed DuraNet Plus	Unwashed YAHE 4.0	0.80	0.54–1.20 (0.290, −1.06)	0.76	Non-inferiority	Non-inferiority not shown
	Washed DuraNet Plus	Washed YAHE 4.0	1.24	0.86–1.78 (0.257, 1.13)	0.76	Non-inferiority	Non-inferior and not superior

## Data Availability

The raw data supporting the conclusions of this article will be made available by the authors on request.

## Supplementary Materials

The following supporting information can be downloaded at: https://www.mdpi.com/article/10.3390/tropicalmed11010026/s1. Figure S1: East African style experimental hut (A) and schematic diagram of its set up in this study (B); Table S1: Summary of net samples that were used for chemical analysis; Table S2a: Mean concentration of alpha-cypermethrin in YAHE^®^ 4.0 LLIN; Table S2b: Mean concentration of Piperonyl butoxide in YAHE^®^ 4.0 LLIN; Table S3a: Mean concentration of alpha-cypermethrin in DuraNet^®^ Plus LLIN; Table S3b: Mean concentration of Piperonyl butoxide in DuraNet^®^ Plus LLIN; Table S4: Mean concentration of alpha-cypermethrin in Interceptor^®^ LLIN.

## Abbreviations

AOR	Adjusted odds ratio
CDC	Centers for Disease Control and Prevention
CI	Confidence Interval
EHTs	Experimental Hut Trials
FIC	First in Class
IRS	Indoor residual spraying
ITNs	Insecticide-treated nets
LLINs	Long-lasting insecticidal nets
NIM	Non-inferiority margin
NIMR	National Institute for Medical Research
PAMVERC	Pan African Malaria Vector Research Consortium
PBO	Piperonyl butoxide
RCTs	Randomized controlled trials
WHO	World Health Organization
WHOPES	World Health Organization Pesticides Evaluation Scheme
WHOPQT	World Health Organization Pre-qualification team
