# Supplementary material for: A Non-Inferiority Evaluation of YAHE 4.0, an Alphacypermethrin-PBO Insecticide-Treated Net Against Pyrethroid Resistant Anopheles arabiensis in Experimental Huts in Moshi, North-Eastern Tanzania
Source: Trop Med Infect Dis. 2026 Jan 18;11(1):26. doi: 10.3390/tropicalmed11010026 (PMC12846432; doi:10.3390/tropicalmed11010026)
Supplement: Supplementary file 1 [file tropicalmed-11-00026-s001.zip › Table S1.pdf]

Table S1: Summary of net samples that were used for chemical analysis

LLIN type	LLIN status	No. of pieces to cut	No. pieces to test in	No. of pieces for chemical	Total pieces for chemical
DuraNet Plus	Before washing*	5	5	5	**20**
	After 20 washes– before hut trial*	5	5	5	
	After hut trial - Olyset® Plus unwashed	5	5	5	
	After hut trial - Olyset® Plus washed 20X	5	5	5	
Interceptor® LLIN	Before washing*	5	5	5	**20**
	After 20 washes– before hut trial*	5	5	5	
	After hut trial - Interceptor® unwashed	5	5	5	
	After hut trial - Interceptor® washed 20X	5	5	5	
YAHE 4.0	Before washing*	5	5	5	**20**
	After 20 washes– before hut trial*	5	5	5	
	After hut trial – YAHE 4.0 unwashed	5	5	5	
	After hut trial – YAHE 4.0 washed 20X	5	5	5	
				**Total**	**60**
