# Supplementary material for: A Non-Inferiority Evaluation of YAHE 4.0, an Alphacypermethrin-PBO Insecticide-Treated Net Against Pyrethroid Resistant Anopheles arabiensis in Experimental Huts in Moshi, North-Eastern Tanzania
Source: Trop Med Infect Dis. 2026 Jan 18;11(1):26. doi: 10.3390/tropicalmed11010026 (PMC12846432; doi:10.3390/tropicalmed11010026)
Supplement: Supplementary file 1 [file tropicalmed-11-00026-s001.zip › Table S2a.pdf]

Table S2a: Mean concentration of alpha-cypermethrin in YAHE® 4.0 LLIN

Active substance	Net sample condition (*)	Times washed	Mean content (g/kg) (n = 5)	Variation (RSD) (n = 5)	Retention (relative to content before washing)	Wash resistance index (%)
Alpha-	BHT	0	8.70	4.4%		
Whole net, 120 denier	BHT	20	8.25	1.9%	94.9%	99.7%
	AHT	0	8.33	1.9%		
	AHT	20	7.75	1.1%	93.0%	99.6%

(*) BHT = Before Hut Trial; AHT = After Hut Trial

RSD=Relative Standard Deviation
