# Supplementary material for: A Non-Inferiority Evaluation of YAHE 4.0, an Alphacypermethrin-PBO Insecticide-Treated Net Against Pyrethroid Resistant Anopheles arabiensis in Experimental Huts in Moshi, North-Eastern Tanzania
Source: Trop Med Infect Dis. 2026 Jan 18;11(1):26. doi: 10.3390/tropicalmed11010026 (PMC12846432; doi:10.3390/tropicalmed11010026)
Supplement: Supplementary file 1 [file tropicalmed-11-00026-s001.zip › Table S2b.pdf]

Table S2b: **Mean concentration of Piperonyl butoxide in YAHE® 4.0 LLIN**

Synergist	Net sample condition (*)	Times washed	Mean content (g/kg) (n = 5)	Variation (RSD) (n = 5)	Retention (relative to content before washing)	Wash resistance index (%)
Piperonyl butoxide	BHT	0	3.46	2.5%		
Whole net, 120 denier	BHT	20	3.08	1.1%	88.9%	99.4%
	AHT	0	3.11	1.9%		
	AHT	20	2.73	1.6%	87.7%	99.3%

(*) BHT = Before Hut Trial; AHT = After Hut Trial
RSD=Relative Standard Deviation
