# Supplementary material for: A Non-Inferiority Evaluation of YAHE 4.0, an Alphacypermethrin-PBO Insecticide-Treated Net Against Pyrethroid Resistant Anopheles arabiensis in Experimental Huts in Moshi, North-Eastern Tanzania
Source: Trop Med Infect Dis. 2026 Jan 18;11(1):26. doi: 10.3390/tropicalmed11010026 (PMC12846432; doi:10.3390/tropicalmed11010026)
Supplement: Supplementary file 1 [file tropicalmed-11-00026-s001.zip › Table S3a.pdf]

Table S3a: **Mean concentration of alpha-cypermethrin in DuraNet® Plus LLIN**

Active substance	Net sample condition (*)	Times washed	Mean content (g/kg) (n = 5)	Variation (RSD) (n = 5)	Retention (relative to content before washing)	Wash resistance index (%)
**Alpha-cypermethrin**	BHT	0	6.11	0.8%		
**Whole net, 150 denier**	BHT	20	5.53	1.0%	90.5%	99.5%
	AHT	0	5.91	2.3%		
	AHT	20	5.55	2.5%	93.9%	99.7%

(*) BHT = Before Hut Trial; AHT = After Hut Trial
RSD=Relative Standard Deviation
