# Supplementary material for: A Non-Inferiority Evaluation of YAHE 4.0, an Alphacypermethrin-PBO Insecticide-Treated Net Against Pyrethroid Resistant Anopheles arabiensis in Experimental Huts in Moshi, North-Eastern Tanzania
Source: Trop Med Infect Dis. 2026 Jan 18;11(1):26. doi: 10.3390/tropicalmed11010026 (PMC12846432; doi:10.3390/tropicalmed11010026)
Supplement: Supplementary file 1 [file tropicalmed-11-00026-s001.zip › Table S3b.pdf]

**Table S3b: Mean concentration of Piperonyl butoxide in DuraNet® Plus LLIN**

| Synergist | Net sample condition (*) | Times washed | Mean content (g/kg) (n = 5) | Variation (RSD) (n = 5) | Retention (relative to content before washing) | Wash resistance index (%) |
|---|---|---|---|---|---|---|
| **Piperonyl butoxide** | BHT | 0 | 1.86 | 4.5% | | |
| **Whole net, 150 denier** | BHT | 20 | 1.66 | 3.0% | 89.2% | 99.4% |
| | AHT | 0 | 1.71 | 4.6% | | |
| | AHT | 20 | 1.61 | 3.5% | 93.9% | 99.7% |

(*) BHT = Before Hut Trial; AHT = After Hut Trial
RSD=Relative Standard Deviation
